# An assessment of the factors affecting the commercialization of cell-based therapeutics: a systematic review protocol

**DOI:** 10.1186/s13643-017-0517-4

**Published:** 2017-06-26

**Authors:** David Pettitt, Zeeshaan Arshad, Benjamin Davies, James Smith, Anna French, Doug Cole, Kim Bure, Sue Dopson, David DiGiusto, Jeff Karp, Brock Reeve, Richard Barker, Georg Holländer, David Brindley

**Affiliations:** 1 The Oxford – UCL Centre for the Advancement of Sustainable Medical Innovation (CASMI), The University of Oxford, Oxford, UK; 2 Department of Paediatrics, University of Oxford, Oxford, UK; 3 University of St. Andrews School of Medicine, University of St. Andrews, St. Andrews, UK; 4 Docherty Gardens, Glenrothes, KY7 5GA UK; 5 Nuffield Department of Orthopedics, Rheumatology and Musculoskeletal Sciences, University of Oxford, Oxford, UK; 6 Flagship Ventures, Cambridge, USA; 7 Sartorius Stedim, Göttingen, Germany; 8 Said Business School, University of Oxford, Oxford, UK; 9 Division of Cell Transplantation and Regenerative Medicine, University of Stanford, Stanford, USA; 10 Harvard Medical School, Harvard University, Boston, USA; 11 Brigham and Women’s Hospital, Boston, USA; 12 Harvard-MIT Division of Health Sciences and Technology, Cambridge, USA; 13 Harvard Stem Cell Institute, Cambridge, USA; 14 Department of Biomedicine, University of Basel and Basel University Children’s Hospital, Basel, Switzerland; 15 Centre for Behavioral Medicine, UCL School of Pharmacy, University College London, London, UK; 16 USCF-Stanford Center of Excellence in Regulatory Science and Innovation (CERSI), Stanford, USA; 17 Orthopedic Surgery Departement, University of Cambridge, Cambridge, UK

**Keywords:** Cell-based therapies, Translational medicine, Commercialization, Clinical adoption

## Abstract

**Background:**

Cellular-based therapies represent a platform technology within the rapidly expanding field of regenerative medicine and are distinct from conventional therapeutics—offering a unique approach to managing what were once considered untreatable diseases. Despite a significant increase in basic science activity within the cell therapy arena, alongside a growing portfolio of cell therapy trials and promising investment, the translation of cellular-based therapeutics from “bench to bedside” remains challenging, and the number of industry products available for widespread clinical use remains comparatively low. This systematic review identifies unique intrinsic and extrinsic barriers in the cell-based therapy domain.

**Methods/design:**

Eight electronic databases will be searched, specifically Medline, EMBASE (OvidSP), BIOSIS & Web of Science, Cochrane Library & HEED, EconLit (ProQuest), WHOLIS WHO Library Database, PAIS International (ProQuest), and Scopus. Addition to this gray literature was searched by manually reviewing relevant work. All identified articles will be subjected for review by two authors who will decide whether or not each article passes our inclusion/exclusion criteria. Eligible papers will subsequently be reviewed, and key data extracted into a pre-designed data extraction scorecard. An assessment of the perceived impact of broad commercial barriers to the adoption of cell-based therapies will be conducted. These broad categories will include manufacturing, regulation and intellectual property, reimbursement, clinical trials, clinical adoption, ethics, and business models. This will inform further discussion in the review. There is no PROSPERO registration number.

**Discussion:**

Through a systematic search and appraisal of available literature, this review will identify key challenges in the commercialization pathway of cellular-based therapeutics and highlights significant barriers impeding successful clinical adoption. This will aid in creating an adaptable, acceptable, and harmonized approach supported by apposite regulatory frameworks and pertinent expertise throughout the respective stages of the adoption cycle to facilitate the adoption of new products and technologies in the industry.

**Electronic supplementary material:**

The online version of this article (doi:10.1186/s13643-017-0517-4) contains supplementary material, which is available to authorized users.

## Review background

As a key constituent of regenerative medicine, cell-based therapies are an exciting platform technology with the prospect of potentially curing what were once considered untreatable diseases [1]. Defined by the BSI (British Standards Institute) as “the therapeutic application of cells regardless of cell type or clinical indication” [2], they present a scientifically, commercially, and clinically important treatment modality. They are distinct from conventional pharmaceuticals, biologics, and medical devices through their capacity to facilitate the de novo production of functional tissue [2] and potential ability to remedy, rather than ameliorate, a spectrum of medical and surgical diseases. Consequently, this has made them attractive to a number of stakeholders, including healthcare practitioners, industry, and investors—all of whom are driving a paradigm shift away from conventional disease management [3].

The cell therapy industry centers on the notion of therapeutically utilizing cells across a multiplicity of disease indications, spanning diverse fields such as neurology, cardiology, and ophthalmology, and extending to both chronic and acute disease states [4, 5]. Despite its relative infancy, it has successfully established itself as a billion-dollar industry [3, 6], which is projected to grow—supported by an increasing number of marketable products, fiscal investment, and strong M&A (merger and acquisition) activity [7]. In 2014 alone, venture capital investment into the biotech sector surpassed $9 billion (USD) [8] and transformed an industry historically plagued by overly exuberant investments and multibillion-dollar losses [9] into a stable and sustainable market that appears attractive for both future investment and long-term value.

### Purpose

This systematic review will critically examine key challenges in the commercialization pathway of cellular-based therapeutics and highlights significant barriers impeding successful clinical adoption. This will help formulate an adaptable and harmonized approach to commercialization that is aligned with appropriate regulatory frameworks, whilst providing evidence-based recommendations for the adoption cycle. Ultimately, this will help expedite the availability of efficacious medical treatments to patients with high, unmet clinical needs.

### Previous reviews and rationale

**Fig. 1:**
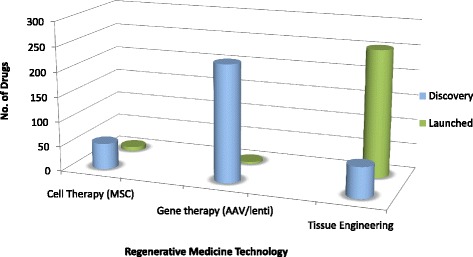
The current regenerative medicine landscape. Compiled using Thomson Reuters Cortellis™ competitive intelligence software. Gene therapy candidates currently lead the *Discovery* phase, whilst most *Launched* drugs comprise tissue-engineered products (including skin substitutes). MSC: mesenchymal stem cell, AAV: adeno-associated virus

Although most cell therapies are currently in pre-launch discovery phases (see Fig. 1), a number of products have achieved success across EU (European Union), US (United States), and global marketplaces. However, despite a promising global demand and favorable investment, the translation of cellular-based therapeutics from “bench to bedside” remains challenging. Difficulties pertaining to healthcare translation are nothing new—the 2006 *Cooksey Report* [10] identified two major translational gaps in health research, namely translating basic science and clinical research into ideas and products, and subsequently introducing these into clinical practice—both of which are relatable to cell therapies. Translational difficulties, and in particular, the clinical adoption of a therapeutic agent, encompass a complex series of processes and relationships between heterogeneous stakeholder groups.

There are a number of review published that look to assess barriers to the development of cell-based therapies. An example is a study conducted by Dodson et al. (2015), who retrospectively analyzed the development of seven cell-based therapies [11]. They concluded that funding, regulation, lack of scientific understanding, reimbursement, and manufacturing are key areas dampening the development of such technology. To the best of our knowledge, however, no systematic evaluation has been conducted to assess the barriers to the commercialization of cell-based therapies. This is a need addressed within this study and is essential to remove bias that may exist when evaluating isolated technologies. Qualitative studies have been previously conducted will also lay the foundation for our pilot data extraction sheet.

## Methods/design

This systematic review will be reported following the Preferred Reporting Items for Systematic Reviews and Meta-Analyses (PRISMA) guidelines [12]. Because this review will only use publically available information, an ethics review board approval will not be required.

### Eligibility criteria

English language manuscripts published within the last 5 years will be included in this review. Inclusion and exclusion criteria to be used are listed in Table 1.

**Table 1:** Inclusion and exclusion criteria

Inclusion criteria	Exclusion criteria
• Published within the last 5 years • English language publications • Addressed regenerative medicines o Autologous or allogeneic • Identified potential challenges in the commercialization or clinical adoption process	• Exclusive focus on manufacturing • Technical papers examining isolation techniques, drug delivery systems or bioprocessing practices • Non-human or veterinary focus • Conference abstracts • Book chapters • Competing interests—sponsored by manufacturer

### Search strategy and search term development

A review of the literature will be conducted to identify published studies from the following bibliographic databases: Ovid MEDLINE, Ovid EMBASE, Cochrane Library, EconLit, BIOSIS, WHOLIS, PAIS International, and Scopus. Due to social science work within the area, a manual review of relevant journals will also be carried out.

The search strategy will be developed using keywords and controlled vocabulary terms (e.g., National Library of Medicine’s Medical Subject Headings). Additional papers will be obtained through the use of citation-tracking software, pursuing bibliographical references of papers electronically identified in the database searches and through further exploration of gray literature (Tables 2 and 3).

**Table 2:** Database search summary

Database	Results (*n* =)	Selected (*n* =)
MEDLINE (OvidSP)	145	62
EMBASE (OvidSP)	1580	194
Cochrane Library & HEED	104	0
EconLit (ProQuest)	296	29
BIOSIS & Web of Science (Thomson Reuters)	159	37
WHOLIS, WHO Library Database	12	0
PAIS International, ProQuest	12	8
Scopus	1181	147
Gray literature/hand search	–	31
Subtotal (*n* =)	3489 (3520 inc gray)	508
Duplicates		146
Total		362

**Table 3:** MeSH terms

	Concept 1	Concept 2	Concept 3	Concept 4	Concept 5
Subject heading	Clinical adoption MeSH: *Technology assessment, Biomedical, Cost-Benefit Analysis*	Cell-based therapeutics MeSH: Cell- and Tissue-Based Therapy, MeSH: biological therapy, MeSH: Regenerative Medicine	Conventional therapeutics MeSH: Therapeutics, Drug Therapy, Enzyme therapy, Molecular Targeted Therapy, Immunotherapy, Transplantation, Monoclonal Antibodies, Vaccines, biosimilars, small-molecule drugs	Emerging technologies MeSH: technology assessment, Biomedical, High-Cost Technology	Barriers
Keywords	Clinical adoption, implementation, technology assessment, appraisal, tools, methodology, commercialization	Biologics, gene therapy, regenerative medicines, cell- and tissue-based therapy, stem cells, tissue engineering	Medicines, pharmacological agents, organ transplant, monoclonal, vaccine	Translational medical research	

Key data regarding electronic database and Thomson Reuters Cortellis™ searches is provided below.

The above electronic databases were first accessed on 17 November 2015.

Key words and medical subheadings:

Subheadings: *cell therapy; biomedical technology assessment; cost benefit analysis; financial management; economic aspect; commercial phenomena; biological therapy; cell therapy; cost; healthcare cost; stem cell; regenerative medicine*

Key words: *commercialization; cell therapy; reimbursement; barrier; regenerative medicine*

Thomson Reuters Cortellis™ Competitive Intelligence Software:

This software will be searched to identify cell therapy products from 01/01/2010 to 01/05/2016 (access date: 01/05/2016). Identified products will be stratified by phase, i.e., launched (in market) or in discovery phase.

Key search terms: *cell therapy; gene therapy (limited to AAV and lentivirus); mesenchymal stem cell*

### Study selection

Two independent reviewers will screen manuscript titles and abstracts for relevance, and full text papers will be obtained for further citations deemed potentially appropriate. Reviewer discrepancies will be discussed until consensus is reached. Full text papers will then be assessed for eligibility according to predefined inclusion and exclusion criteria (as listed in Table 1).

**Fig. 2:**
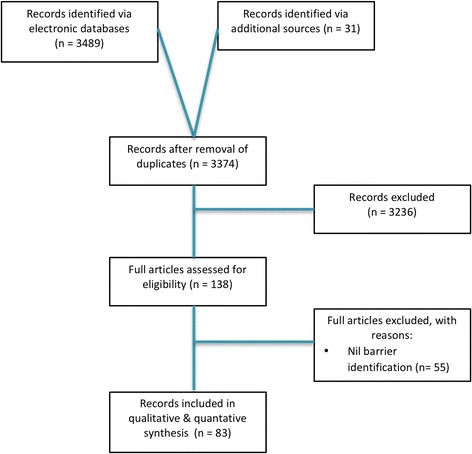
Systematic review methodological overview

Eligible papers will be subsequently reviewed and key data extracted into a pre-designed data extraction scorecard. An overview of the methodology can be seen in Fig. 2 below:

### Synthesis

The data synthesis and extraction scorecard will be categorized into eight key domains, which are outlined below:

1. Manufacturing
2. Regulation and intellectual property
3. Reimbursement
4. Clinical Trials
5. Clinical Adoption
6. Ethics
7. Business models
8. Other

Such domains were identified as being key through brief review of the literature in the area. Each domain will be further subcategorized into important components, e.g., within the *Manufacturing* domain, subcategories will include *Scalability*, *Automation*, and *Supply Chain*. The tabulated scorecard serves to:

i. Facilitate the assignment of a *perceived impact and/or importance* score
ii. Serve as a record of frequency for which a barrier/domain was mentioned in a manuscript

The data synthesis and extraction scorecard will be piloted on a sample of 23 manuscripts and completed by two independent reviewers. The pilot sample will be acquired from the 83 records identified by stratifying them according to *highest impact factor journal* (*n =* 13) and *number of paper citations* (*n =* 10). The scorecard will subsequently be applied to all 83 identified records and completed by two independent reviewers. The IRR (inter-rater reliability) will be calculated following analysis.

The data synthesis and extraction scorecard will score each paper on the perceived impact and importance of a cited factor, as seen below in Table 4.

**Table 4:** Data synthesis and extraction scorecard excerpt

Perceived impact and importance			
Essential (4)	Major (3)	Moderate (2)	Negligible (1)
Imperative barrier, definitive consideration	High priority, key consideration	Lower value or narrower impact	Low priority or relevance to field, minimal impact

The excerpt above displays a linear scale ranging from *Essential* to *Negligible*, which is accompanied by a numerical value (see parenthesis). The scorecard will also facilitate the documentation of additional information, including publication details (e.g., year, journal, country of publication), cell-based therapy characteristics (e.g., therapeutic indication, autologous vs. allogeneic, cell type), generalizability (e.g., if nature of findings are limited to a particular region by the regulatory system described), and sources of funding and potential conflicts of interest. An additional free text box will be included for “other” challenges or barriers identified in the reviewed manuscripts that did not fall into one of the predetermined domains or subcategories.

A PRISMA-P file is included as Additional file 1. There is no PROSPERO registration number.

## Discussion

This systematic review will critically examine key challenges in the commercialization pathway of cellular-based therapeutics and highlights significant barriers impeding successful clinical adoption. This will help formulate an adaptable and harmonized approach to commercialization that is aligned with appropriate regulatory frameworks, whilst providing evidence-based recommendations for the adoption cycle. Ultimately, this will help expedite the availability of efficacious medical treatments to patients with high, unmet clinical needs.

A number of limitations will be inherent to this study. Notably, the assignment of an impact score is subjective and open to reviewer interpretation. Publication bias is also inherent to the academic literature and it is plausible that more important or challenging commercialization barriers are more widely discussed and, consequently, published. Challenges are also experience-dependent. Cell therapy developers may therefore have a greater degree of real-world experience with the initial phases of commercialization, e.g., manufacturing or seeking regulatory approval with demonstrable clinical trial data, in comparison to the latter phases, such as clinical adoption or reimbursement.

Due to the rapid evolution of the regenerative medicine field, it was deemed appropriate to only include papers published in the last 5 years. It is however likely that a number of studies published at the beginning of this period are now outdated. It may also be possible that a number of relevant studies published prior to this time period contain valid arguments for ongoing commercialization challenges and may have been missed. The study was also limited to English language publications, which may have resulted in research findings, particularly from South East Asian nations with novel regulatory mechanisms, being excluded. Such limitations can be mitigated in future research that employs more extensive inclusion criteria and regularly re-visits key literature sources.

## Additional file

Additional file 1: PRISMA-P (Preferred Reporting Items for Systematic review and Meta-Analysis Protocols) 2015 checklist: recommended items to address in a systematic review protocol*. (DOC 81 kb)

## Abbreviations

IRR: Inter-rater reliability
PRISMA: Preferred Reporting Items for Systematic Reviews and Meta-Analyses
